# COMPONENTS OF FRONTAL ASSESSMENT BATTERY AND CLINICAL FEATURES IN PATIENTS WITH STROKE

**DOI:** 10.2340/jrm.v57.43270

**Published:** 2025-06-25

**Authors:** Katsuya SAKAI, Yuichiro HOSOI, Yusuke HARADA, Yuichi KATO, Takayuki MIYAUCHI

**Affiliations:** 1 Department of Physical Therapy, Faculty of Health Sciences, Tokyo Metropolitan University, Tokyo; 2 Department of Rehabilitation of Medicine, Keio University School of Medicine, Tokyo; 3 Department of Rehabilitation, Reiwa Rehabilitation Hospital, Chiba; 4 Department of Rehabilitation, Moriyama Neurological Center Hospital, Tokyo; 5 Department of Rehabilitation, Faculty of Medical Sciences, Shonan University of Medical Sciences, Kanagawa, Japan

**Keywords:** Frontal Assessment Battery, executive functions, principal component analysis, cluster analysis, clinical features

## Abstract

**Objective:**

First, to investigate whether the 6 aspects of executive functions assessed by the Frontal Assessment Battery have different components and, if so, to extract those components using principal component analysis. Second, to identify patient groups based on their characteristics using cluster analysis.

**Design:**

A cross-sectional study.

**Subjects:**

Seventy-eight patients with stroke.

**Methods:**

The Frontal Assessment Battery, Mini-Mental State Examination, Trail Making Test, and Stroop Color Word Test were performed within 5 days.

**Results:**

Based on principal component analysis, the Frontal Assessment Battery was classified into cognitive control (subscales 1–3, 5) and behavioural control (subscales 4 and 6). Two clusters that reflect these components (cluster 1, *n* = 68; cluster 2, *n* = 10) were identified. The between-group comparison showed that compared with cluster 1, cluster 2 had lower scores on Frontal Assessment Battery subscales 4 and 6, the Frontal Assessment Battery total scores, and other executive functions scores. The Mini-Mental State Examination scores had no significant differences.

**Conclusions:**

The Frontal Assessment Battery can be classified into 2 components, and the impairment of Frontal Assessment Battery subscales 4 and 6 identified a specific group of patients with stroke with severe executive dysfunction.

## LAY ABSTRACT

The Frontal Assessment Battery evaluates executive functions using 6 subscales. This study investigated whether the 6 subscales of FAB assessed 6 components of executive functions in patients with stroke. A total of 78 stroke patients participated in this observational study. As a result, it was divided into 2 components (group 1, *n* = 68; group 2, *n* = 10). The between-group comparison showed that compared with group 1, group 2 had lower scores on Frontal Assessment Battery subscales 4 and 6, Frontal Assessment Battery total scores, and other executive functions scores. The Frontal Assessment Battery was classified into intelligent motor programming type (items 1–3, 5), and behaviour and environmental type (items 4 and 6). The Frontal Assessment Battery can be classified into 2 components, and the impairment of Frontal Assessment Battery subscales 4 and 6 identified a specific group of patients with stroke with severe executive dysfunction.

Executive functions are high-level brain functions that include decision-making, risk-taking, planning, inhibitory control, working memory, and cognitive flexibility (1). Executive and cognitive functions are important in the daily lives of older adults and patients with stroke (2, 3). However, 25–75% of patients with stroke suffer from executive dysfunction (1, 4). Executive dysfunction, in particular stroke-induced executive dysfunction, causes a decline in activities of daily living (ADL) and instrumental ADL function, and also limits social reintegration (5–7). In addition, the risk of admission to a facility increases with the decline in executive functions (inhibition control) caused by stroke (8), and the rate of return-to-work is limited because of the decline in executive functions in patients with stroke (6). Therefore, focusing on executive dysfunction in patients with stroke is important.

Several tools are available for assessing executive dysfunction caused by stroke (9), including the Frontal Assessment Battery (FAB) (10), Trail Making Test (TMT) (11), Behavioural Assessment of Dysexecutive Syndrome (BADS) (12), Stroop Color and Word Test (SCWT) (13), and Tower of London (TL) (14). The TMT, SCWT, and TL are screening tests that can be used to briefly assess executive functions (9); however, evaluating executive functions separately is difficult. These assessments include those of execution functions of the BADS and FAB, conducted by dividing it into several components. The FAB assesses executive functions using several components (10), the advantage of which is that it can briefly assess and divide executive functions into 6 categories: conceptualization, mental flexibility, motor programming, conflicting instructions, inhibitory control, and environmental autonomy, each of which can be assessed subscale-wise. Assessing executive functions by components is important for considering rehabilitation strategies, because executive functions include many different components (15, 16), and rehabilitation methods differ depending on which components are impaired (9). However, whether the 6 FAB subscales actually assess different aspects of executive functions is unclear in patients with stroke; if they do, we assume that they could be classified into categories. Additionally, we assume that if different components comprise the FAB, then there would be patient groups that characterize each component (e.g., patients with impaired inhibitory control and patients with impaired environmental autonomy). Wang et al. used principal component analysis (PCA) to investigate whether the FAB could be classified into components using a sample of healthy participants (17). The results revealed that the FAB could be classified into either 2 or 3 principal components. In the first solution, the 2 principal components are cognitive control (mental flexibility, conceptualization, inhibitory control) and behavioural control (sensitivity to interference, environmental autonomy, motor programming). In the second solution, the 3 principal components are automatic behavioural control (sensitivity to interference, environmental autonomy), cognitive control (mental flexibility, conceptualization), and monitored behavioural control (motor programming, inhibitory control). Therefore, we used PCA to investigate the elements that comprise the 6 FAB subscales according to the previous study (17). We then classified the data using cluster analysis to identify groups of patients along the elements and to then extract patient characteristics. By examining these distinct characteristics, this study suggests that the FAB should be evaluated or analysed not only by total score but also relative to specific subscales, and that rehabilitation should be provided in accordance with impaired components of executive functions in combination with other assessments. This study aimed to clarify the FAB components and to identify patient groups based on their characteristics.

## MATERIALS AND METHODS

### Participants

Participants comprised 78 patients with stroke (age: mean = 67.8 [± 12.6] years; sex: male 46, female 32; body mass index [BMI]: mean 22.2 [SD 3.7] kg/m^2^; time since stroke: mean = 54.1 [± 53.3] days; modified Rankin Scale [mRS]: median 5). The inclusion criteria were: (1) first-time stroke, (2) age > 18 years, (3) presence of hemiplegia, and (4) supratentorial lesions diagnosed by a neurological doctor. The exclusion criteria were as follows: (1) diagnosis of severe dementia and Alzheimer’s disease, (2) diagnosis of higher brain dysfunction (e.g. unilateral spatial neglect, aphasia, and apraxia), and (3) age > 90 years. Patients received an explanation of the study’s purpose, after which written informed consent was obtained prior to study initiation. This study was approved by the ethics committee of the Ishikawajima Memorial Hospital, Reiwa Rehabilitation Hospital, and Moriyama Neurological Center Hospital (approval number: 2022-02, 00-10, and 23002, respectively). It is publicly registered in the UMIN Clinical Trials Registry (UMIN-CTR) (trial registration ID: UMIN000048587), and complies with the ethical standards established in the 1964 Declaration of Helsinki.

### Design and instruments

This study employed a cross-sectional design. All assessments were measured within 5 days. Assessments comprised the Mini-Mental State Examination (MMSE) to assess cognitive function, FAB to assess frontal and executive functions, Trail Making Test (TMT) A and B to assess attention and executive functions, Stroop Color and Word Test (SCWT) Parts I to IV to assess executive functions, and Functional Independence Measure (FIM) cognitive part to assess cognitive function.

The MMSE was used to assess cognitive function, with a total score of 30 points or higher indicating good cognitive function (18). MMSE scores of 0–10, 11–20, 21–23, and ≥ 24 indicated severe cognitive impairment, moderate cognitive impairment, mild cognitive impairment, and normal cognition, respectively (18).

The FAB comprises 6 subscales, each scored from 0 to 3 points: subscale 1, similarities, assesses conceptualization; subscale 2, lexical-verbal fluency, assesses mental flexibility; subscale 3, motor series, assesses programming; subscale 4, conflicting instructions, assesses sensitivity to interference; subscale 5, go/no-go, assesses inhibitory control; and subscale 6, prehension behaviour, assesses environmental autonomy. The maximum total score is 18 points, with higher scores indicating better frontal lobe and executive functions (10).

TMT part A reflects motor speed and attention function, whereas part B is often used to assess executive functions (11). For TMT part A, the participants connected circled numbers (from 1 to 25) in sequence as quickly as possible (11). In TMT part B, participants connected numbers and letters alternately in sequence as quickly as possible (11).

The SCWT can assess executive functions, and comprises 4 parts (13). SCWT parts I to IV are each 40-s tests, and they comprise 30 pieces. In Part I, participants select colour words written in black ink and the matching colour. In part II, participants select colour words written in various colours and the matching colour. In part III, participants select colour words written in various colours and black ink, and in part IV, they select colour words written in various colours and the corresponding colour words written in black ink. The SCWT calculates the correct and incorrect answer, and the number of correct answers is used as a measure of executive functions (13).

The FIM assessments measure the ability to perform ADL, and comprise the FIM motor items (13 items) and FIM cognitive items (5 items) (19). In particular, FIM cognitive items comprise communication (expression and comprehension) and social cognition (social interaction, problem solving, and memory), and they can be used to assess the cognitive functions necessary for daily life. Each item is rated on a 6-point scale (score range, 1–7), with 91, 35, and 126 being the highest scores for the FIM motor, cognitive, and total items, respectively. Higher scores indicate better ADL functioning. Only FIM cognitive items were used in this study.

### Statistical analysis

To extract the FAB components, this study conducted a PCA using subscales 1–6 of the FAB. Consistent with a previous study (20), the current investigation set the eligibility criteria for the eigenvalues of the principal components (PC) at more than 1.0, and determined that items with correlation values of more than 0.4 were related. Cluster analysis using the word method was conducted using each participant’s PC 1 and 2 values. The number of clusters was determined *a priori* based on the PCA results. These values were also used to classify patients into groups. Subsequently, the groups were compared based on the number of clusters, and the groups were classified into 2 clusters and compared using the Mann–Whitney *U* test. χ^2^ tests were used for sex, type of stroke, and paretic side. Statistical analysis was performed using SPSS (version 29.0; IBM Corp, Armonk, NY, USA), and the statistical significance was set at *p* < 0.05.

## RESULTS

A total of 78 patients with stroke (age: mean 67.8 [SD 12.6] years; time since stroke: mean 54.1 [SD 53.3] days, mRS: median 5, **Table I**) were considered. The values for each subscale in the FAB, the total score, and other variables are listed in **Table II**.

**Table I:** Basic attributes of participants

Variables	Overall (*n* = 78)	Cluster 1 (*n* = 68)	Cluster 2 (*n* = 10)	*p*-value
Age, years, mean ± SD (range)	67.8 ± 12.6 (41–89)	67.8 ± 12.6 (41–89)	67.6 ± 13.1 (47–84)	0.988
Sex (male/female), *n* (%)	46/32 (59.0%/41.0%)	39/29 (57.4%/42.6%)	7/3 (70%/30%)	0.513
BMI, kg/m^2^, mean ± SD (range)	22.2 ± 3.7 (15.6–33.5)	22.3 ± 3.8) (15.6–33.5)	21.5 ± 3.1 (16.2–25.9)	0.622
mRS, median (range)	5 (3–5)	5 (3–5)	5 (3–5)	0.651
Type of stroke (infarction/haemorrhagic), *n* (%)	40/38 (51.3%/48.7%)	36/32 (52.9%/47.1%)	4/6 (40%/60%)	0.335
Paretic side (right/left), *n* (%)	38/40 (48.7%/51.3%)	38/30 (55.9%/44.1%)	2/8 (20%/80%)	0.114
Time since stroke, days, mean ± SD (range)	54.1 ± 53.3 (2–414)	47.8 ± 34.3 (2–125)	97.0 ± 115.0 (30–414)	0.045

BMI: body mass index; mRS: modified Rankin Scale. Mann–Whitney *U* tests and χ^2^ tests were used for statistical analysis.

**Table II:** Results of FAB scores and other assessments scores

Variables	Overall (*n* = 78)	Cluster 1 (*n* = 68)	Cluster 2 (*n* = 10)	Z-value	*p-*value
FAB 1	3 (0–3)	3 (0–3)	2.75 (2–3)	–0.20	0.838
FAB 2	2 (0–3)	2.25 (0–3)	1.75 (1–3)	–1.89	0.059
FAB 3	3 (0–3)	3 (0–3)	2 (2–3)	–1.01	0.311
FAB 4	3 (1–3)	3 (1.5–3)	2.25 (1–3)	–4.37	< 0.001
FAB 5	2.5 (0–3)	2.5 (0–3)	2 (0.5–3)	–1.55	0.121
FAB 6	3 (2–3)	3 (3–3)	2 (2–3)	–7.18	< 0.001
FAB total scores	14.8 (2.5)	15.2 (2.6)	13.0 (1.1)	–2.95	0.003
MMSE	27.8 (2.3)	27.9 (2.3)	27.3 (2.2)	–0.93	0.35
TMT part A	61.4 (28.2)	58.2 (26.4)	83.1 (31.5)	–2.43	0.015
TMT part B	150.3 (127.7)	137.2 (126.4)	239.4 (102.0)	–3.4	0.001
SCWT part I	22.3 (7.9)	23.1 (8.0)	16.5 (4.6)	–2.55	0.011
SCWT part II	20.1 (7.5)	20.8 (7.5)	14.9 (5.7)	–2.38	0.017
SCWT part III	18.2 (7.2)	18.8 (7.1)	13.6 (4.8)	–2.09	0.037
SCWT part IV	13.7 (7.4)	14.4 (7.3)	9.2 (6.5)	–1.98	0.048
FIM cognitive	28.9 (6.9)	28.7 (6.8)	30.0 (5.2)	–0.14	0.886

Data are expressed as mean (standard deviation) or median (min–max).

FAB: Frontal Assessment Battery; MMSE: Mini-Mental State Examination; TMT: Trail Making Test; SCWT: Stroop Color and Word Test; FIM: Functional Independence Measure. Mann–Whitney *U* tests were used for statistical analysis.

The PCA results revealed 2 PCs with eigenvalues greater than 1.0 (PC1: 2.36; PC2: 1.13). The cumulative rate of PC1 was 58.2% (variance 39.4%) and of PC2 was 58.2% (variance 18.8%). The correlation matrix heat map is presented in **Table III**. PC1 comprised FAB subscales 1, 2, 3, and 5; the correlation value of this group was more than 0.4. Based on the characteristics of the subscales, it was defined as cognitive control. PC2 comprised FAB subscales 4 and 6; the correlation value of this group was more than 0.4. Based on the characteristics of the subscales, it was defined as behavioural control.

**Table III:** Heatmap and correlation value of PC1 and PC2

	PC1	PC2
FAB 2 subscale	**0.787**	0.011
FAB 5 subscale	**0.750**	–0.125
FAB 3 subscale	**0.734**	–0.221
FAB 1 subscale	**0.685**	–0.212
FAB 6 subscale	0.225	**0.817**
FAB 4 subscale	0.352	**0.592**

PC: principal components; FAB: Frontal Assessment Battery. Bold numbers indicate PC grouping.

**Fig. 1:**
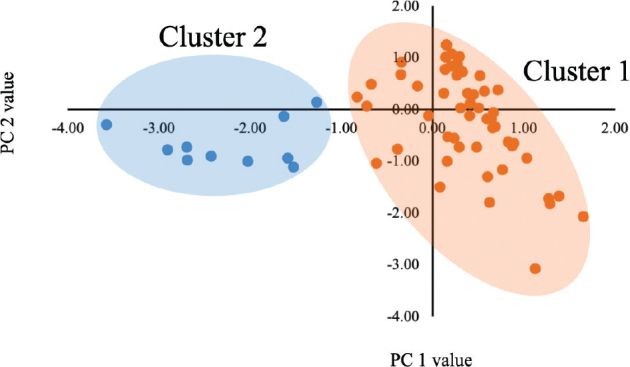
Scatter plot of principal component values and cluster.

Following the cluster analysis, the PC1 and PC2 data were classified into 2 clusters (cluster 1: *n* = 68, 87.2%; cluster 2: *n* = 10, 12.8%) according to their values (**Fig. 1**). Time since stroke in cluster 2 was significantly longer than in cluster 1 (Z = –2.00, *p =* 0.045; see Table I). However, age, sex, BMI, stroke type, paralysis side, or disease severity had no significant differences (*p* > 0.05, Table I). Subscales 4 and 6 as well as total FAB scores were significantly lower in cluster 2 than in cluster 1 (FAB 4 subscale: Z = –4.37, *p <* 0.001; FAB 6 subscale: Z = –7.18, *p <* 0.001; FAB total scores: Z = –2.95, *p =* 0.003; see Table II). TMT part A, B, and SCWT parts I to IV in cluster 2 were significantly different from those in cluster 1 (TMT part A: Z = –2.43, *p =* 0.015; TMT part B: Z = –3.4, *p =* 0.001; SCWT part I: Z = –2.55, *p =* 0.011; part II: Z = –2.38, *p =* 0.017; part III: Z = –2.09, *p =* 0.037; part IV: Z = –1.98, *p =* 0.048; Table II). However, there were no significant differences in FAB items 1, 2, 3, 5, MMSE, and FIM cognitive scores (*p* > 0.05; Table II).

## DISCUSSION

This study extracted the components of FAB using PCA, conducted cluster analysis using these PC values, and analysed the patients’ executive functions characteristics. Consequently, the FAB comprised 2 components (cognitive control, behaviour control) and identified a specific patient group with severe executive functions impairment, in which FAB subscales 4 and 6 were impaired. This study suggests that the FAB should be used to assess total scores, as well as the stroke patients’ features by subscale, and that rehabilitation should be provided according to the impaired elements of executive functions combined with other assessments.

The PCA results classified the FAB into 2 components: subscales 1, 2, 3, and 5; and subscales 4 and 6. The PCA results align with previous studies (17, 21). As discussed earlier, based on PCA results, Wang et al. (17) divided the FAB into either 2 or 3 components. Similarly, Dubois et al. (21) reported that FAB subscales 1, 2, 3, and 5 respectively assess abstract thinking; verbal fluency; time organization, maintenance, and motor learning; and inhibition control. Each of these subscales entails thinking assignments in response to instructions and the necessary tasks for the given cognitive control. Therefore, it was assumed that these subscales could be classified into the same component. Conversely, FAB subscale 4 assesses the self-regulation of behaviour and response-selection tasks, and subscale 6 assesses dependence on environmental cues. As these assessments are related to behaviour and the environment, we assumed that these subscales could be classified as 1 component. Kopp et al. (22) identified brain regions associated with the total FAB score and each FAB subscale. Consequently, they reported that the total FAB score, FAB conceptualization score (subscale 1), and FAB inhibitory control score (subscale 5) were associated with the anterior insula (BA13) in patients with stroke. Furthermore, their study reported that the FAB mental flexibility score (subscale 2) was associated with damage to the middle frontal gyrus (BA9) and that the FAB inhibitory control score (subscale 5) was associated with damage to the right inferior frontal gyrus (BA44/45) in patients with stroke. Interestingly, FAB programming (subscale 3), FAB interference susceptibility (subscale 4), and FAB environmental autonomy scores (subscale 6) were not associated with frontal lobe lesions (21). Similar to the findings of Kopp et al. (22), our PCA analysis results classified FAB subscales 4 and 6 as different components. However, other studies have reported lower total FAB scores in patients with frontal lobe lesions (23). In addition, executive functions are not limited to the frontal lobe but are connected to various regions in a network (24). Additionally, frontal lobe symptoms appear even when areas other than the frontal lobe are affected (25). Therefore, we did not obtain consistent results, nor did we assess the relationship between brain regions and each subscale on the FAB in this current study. Hence, further studies are required to clarify these issues.

Cluster 2 had significantly lower FAB subscales 4 and 6, lower total FAB scores, TMT parts A and B, and SCWT parts I to IV scores, and longer time since stroke compared with cluster 1. However, cognitive function (MMSE and FIM cognitive scores) exhibited no significant differences. As mentioned earlier, FAB subscale 4 assesses sensitivity to interfering stimuli and response selection tasks, whereas subscale 6 assesses environmental autonomy, which comprises reactions to behaviour and the environment. The response selection to be assessed in subscale 4 was similar to the task selection included in TMT parts A and B. FAB subscale 4 requires either 1 tap or 2 taps (10, 21). Therefore, the number of taps is selected based on the task. Similarly, TMTs are connected by selecting the next number to be connected in part A, whereas in part B, the numbers and letters are selected (11). We speculated that low scores on FAB subscale 4 would also be associated with the TMT, which assesses these selection abilities. Furthermore, TMT and FAB are associated with SCWT (10, 26). Thus, we assumed that cluster 2, with its severe FAB subscale 4 scores, would have lower attention and executive functions than cluster 1. FAB subscale 6 was easier than the other items, because it had a ceiling effect not only in mild cognitive impairment (26) but also in stroke (10, 23) and Parkinson’s disease (28). In other words, subscale 6 is not difficult in any disease and other FAB subscales. Despite the lower difficulty of subscale 6, cluster 2 of this subscale had significantly lower scores than cluster 1. Therefore, we assumed that cluster 2 had lower executive functioning than cluster 1. In addition, the length of time since stroke may be associated with lower behavioural control. Previous studies have reported that severe executive dysfunction is associated with difficulties in ADL, instrumental ADL, and social reintegration (5–7). Therefore, we assumed that time since stroke in cluster 2 was longer than in cluster 1.

This study has several limitations. First, we did not investigate which brain regions were impaired between clusters. Studies have reported that the FAB has different brain regions responsible for each subscale (22, 29). Future studies should address this limitation. Second, we did not assess motor function. Our work focused on higher brain functions to extract the characteristics of executive dysfunction. In a future study, we plan to investigate their effects on motor function. Third, this study had a small sample size and was conducted in only 3 hospitals. However, these findings suggest that this group of patients had specific disorders. Therefore, in future investigations, we will explore this issue with a larger sample size and conduct studies across several hospitals. Finally, because this was a cross-sectional study, the degree of recovery and outcomes for each cluster are unknown. Therefore, longitudinal studies should be conducted in the future.

In conclusion, this study used PCA to extract FAB components and analyse the characteristics of patients with stroke. FAB subscales 4 and 6 were found to enable the identification of stroke patients with severe executive dysfunction.

## Data Availability

Data available on request from the authors.

## Trial details

The study is publicly registered in the UMIN Clinical Trials Registry (trial registration ID: UMIN000048587).
